# Correction: Kosuru, R.; Chrzanowska, M. Divergent Functions of Rap1A and Rap1B in Endothelial Biology and Disease. *Int. J. Mol. Sci.* 2025, *26*, 5372

**DOI:** 10.3390/ijms26188996

**Published:** 2025-09-16

**Authors:** Ramoji Kosuru, Magdalena Chrzanowska

**Affiliations:** 1Versiti Blood Research Institute, P.O. Box 2178, Milwaukee, WI 53201-2178, USA; rkosuru@versiti.org; 2Department of Pharmacology and Toxicology, Medical College of Wisconsin, 8701 Watertown Plank Road, Milwaukee, WI 53226, USA; 3Cardiovascular Research Center, Medical College of Wisconsin, 8701 Watertown Plank Road, Milwaukee, WI 53226, USA

## Text Correction

There was an error in the original publication [1]. Reference [11] (RasGRP3: A central nexus for Ca^2+^ entry and distinct Rap1 and H-Ras signaling pathways in endothelial cells) was included as a manuscript that had been accepted at the time of submission but was subsequently revoked by the publishing journal for reasons unrelated to scientific misconduct. To maintain transparency and accuracy, we are removing the citation and related content.

A correction has been made to Section 1, Paragraph 3:

Corrected paragraph:

Rap1 activity is controlled by guanine nucleotide exchange factors (GEFs), which facilitate the exchange of GDP to GTP and active conformation of Rap1, and GTPase-activating proteins (GAPs), which inactivate Rap1. Epac, the best characterized Rap1 GEF in the endothelium, is directly activated by cyclic adenosine monophosphate (cAMP) and mediates acute Rap1 activation via specific analogs [9,10]. RasGRP3 (CalDAG-GEFIII), a diacylglycerol-sensitive GEF with dual specificity for Ras and Rap1, is expressed in embryonic and angiogenic endothelium and contributes to vascular morphogenesis and diabetes-associated dysfunction [11–14].

A correction has been made to Section 3.2., Paragraph 3:

Corrected paragraph:

Measurements of intracellular calcium in Rap1A- and Rap1B-deficient cells revealed that Rap1A, but not Rap1B, specifically suppresses the SOCE component of calcium signaling. This regulation occurs at the transcriptional level, as Rap1A deletion increases Orai1 expression, enhancing calcium influx. Normalizing Orai1 expression with partial siRNA knockdown restores SOCE to baseline [8]. In Rap1A-deficient endothelial cells, thrombin induced elevated NFAT activation, an effect reversed by calcineurin inhibition. Lipid nanoparticle (LNP)-mediated delivery of siOrai1 normalizes Orai1 expression and reduces NFAT activity and inflammation in inducible VE-cadherin-Cre Rap1A-knockout (Rap1A^i∆EC^) mice [8] (Figure 2). These findings demonstrate that Rap1A serves as a key negative regulator of endothelial SOCE and inflammation, a novel function in endothelial calcium homeostasis [55].

A correction has been made to Section 3.3.2.:

Reference [11] was removed.

A correction has been made to Section 5.1., Paragraphs 3 and 4.

Corrected paragraph:

RasGRP3 (CalDAG-GEFIII) is a member of the RasGRP family GEFs with dual specificity for Ras and Rap1 [11]. Initially identified as a vascular gene responsive to phorbol esters, RasGRP3 is expressed in embryonic and angiogenic endothelium and participates in DAG-sensitive morphogenic signaling pathways [12,13]. It promotes endothelial cell migration and mediates diabetes-induced vascular dysfunction via Ras activation, with its activity driven by elevated DAG levels characteristic of the diabetic environment [12].

Studies in macrophages have shown that RasGRP3 controls their critical functions by acting via Rap1 [14]. However, RasGRP3’s in vivo functions remain incompletely understood, and future studies using endothelial cell-specific RasGRP3-knockout models will be necessary to delineate Ras- versus Rap1-dependent outputs.

A correction has been made to Section 7, Paragraph 4.

Corrected paragraph:

Upstream control of Rap1 is also more complex than previously appreciated. While Epac remains a widely used tool to activate Rap1 via cAMP, new GEFs such as RasGRP3 are emerging as physiological regulators. RasGRP3, a dual-specificity GEF for both Rap1 and Ras, contributes to vascular morphogenesis and diabetes-associated endothelial dysfunction. This signaling integration and disease relevance warrant further mechanistic and functional exploration.

## Error in Figure and Figure Legend

In the original publication, there was a mistake in Figure 2 and the legend for Figure 2 as published. In the original version of Figure 2, RasGRP3 was shown as the upstream activator of Rap1. In the revised figure, we have replaced RasGRP3 with ‘Rap1GEF’ to represent potential upstream guanine nucleotide exchange factors more generally, without specifying a particular molecule. RasGRP3 was mentioned in the original legend, but it has been removed from the corrected figure legend to reflect the updated findings. The corrected Figure 2 and the legend for Figure 2 appear below.

## References

With this correction of removing Reference [11], the original references [77–80] have been updated to [11–14], and the order of some references has been adjusted accordingly.

The authors state that the scientific conclusions are unaffected. This correction was approved by the Academic Editor. The original publication has also been updated.

## Figures and Tables

**Figure 2 ijms-26-08996-f002:**
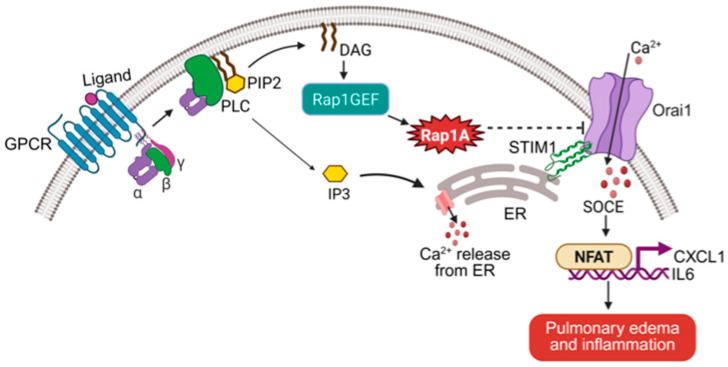
Rap1A restricts Orai1-mediated calcium entry to suppress inflammatory signaling in endothelial cells. The schematic illustrates the Rap1A–calcium signaling axis in endothelial cells. Upon GPCR activation, phospholipase C (PLC) hydrolyzes PIP2 into diacylglycerol (DAG) and inositol 1,4,5-trisphosphate (IP3), a diacylglycerol-sensitive guanine nucleotide exchange factor that activates Rap1A. Activated Rap1A suppresses store-operated calcium entry (SOCE) by limiting Orai1 activation and expression. In the absence of Rap1A, increased calcium influx via Orai1 promotes nuclear translocation of NFAT and transcription of proinflammatory genes such as CXCL1 and IL6, leading to pulmonary edema and inflammation. This pathway identifies Rap1A as a critical negative regulator of calcium-driven endothelial inflammation.
